# In-hospital systems for organ donation after brain death in Japan: A nation-wide survey—advancing brain-dead organ donation systems in Japan—

**DOI:** 10.20407/fmj.2025-007

**Published:** 2025-11-05

**Authors:** Yuuki Takaki, Tomoko Asai, Ayumi Tasaki, Sayuri Nakamura

**Affiliations:** Graduate School of Health Sciences, Fujita Health University, Toyoake, Aichi, Japan

**Keywords:** Brain-dead organ donation, In-hospital organ donation system development, In-hospital donor coordinator, Organ donation option

## Abstract

**Objectives:**

Brain-dead organ donations in Japan remain significantly fewer than in other countries, highlighting inadequacies in the organ donation system. This study aimed to examine the state of organ donation systems in hospitals that have conducted brain-dead organ donations and to explore potential in-hospital improvements.

**Methods:**

Among 148 hospitals that carried out at least one brain-dead organ donation between 2018 and 2023, 84 participated in an anonymous, self-administered online survey, yielding a 57% response rate.

**Results:**

A total of 96% of responding hospitals reported having in-hospital donor coordinators (IHCos), with an average of 7.0±6.0. Their IHCo teams included nurses in 98%, physicians in 50%, and administrative staff in 27% of hospitals. A significant positive correlation was observed between the numbers of organ donation options presented and actual organ donations. Hospitals offering more options tended to have six or more IHCos and/or a system for identifying potential donors. Furthermore, hospitals with full-time IHCos had significantly more deceased organ donors.

**Conclusions:**

Appropriate allocation of IHCos and the establishment of a system to identify potential donors can enhance the assessment of patients’ or their families’ willingness to donate organs and support the successful completion of the donation process.

## Introduction

Brain-dead organ donation became legal in Japan with the 1997 Organ Transplant Act, which required a written declaration of intent from the donor. Following a revision of the Act in 2010, donation with family consent alone became possible when the individual’s intent was unknown, requiring that they had not explicitly refused donation. As a result, the cumulative number of brain-dead organ donations increased from 86 cases before the revision to 1,064 by December 31, 2024, with 843 of those (79.2%) based on family consent.^1^ However, demand has also continued to rise: approximately 16,000 candidates are currently waiting for a transplant, yet only around 600 (about 4%) receive an organ each year.^2^

Under Section 4-3 of the Organ Transplant Act Guidelines, brain-dead organ donations may only take place in advanced emergency medical institutions classified into one of five types: university hospitals, designated training facilities of the Japanese Association for Acute Medicine, core/branch facilities of the Japan Neurosurgical Society, emergency and critical care centers, and member hospitals of the Japanese Association of Children’s Hospitals and Related Institutions. These facilities are referred to here as “five-category hospitals.”

Yet not all of these designated hospitals have adequately established organ donation systems. According to the Ministry of Health, Labour and Welfare,^3^ as of the end of March 2024, there were 906 such hospitals nationwide, but 465 (51%) of them lacked a fully developed system. Of the 906 hospitals, 297 had experience with brain-dead organ donation; among them, 110 hospitals (37.0%) had handled only one case—the most common experience level—while just 17 hospitals (5.7%) had handled 10 or more cases.

The Ministry of Health, Labour and Welfare concluded that because many organ donation hospitals lack experience in brain death determination and end-of-life care, potential brain-dead organ donors may not be properly identified, and families may not receive full and adequate information.^4^ Without systematic support within hospitals, opportunities for donation can be lost even when patients or families would, —in principle—, be willing to donate. Strengthening hospital infrastructure is therefore urgent.

Furthermore, in that study by Health Labour Sciences Research Grant based on data from 612 responding five-category hospitals, the estimated number of deaths for 2023 following a condition that could be considered brain death was 4,412. Of these, 1,363 cases were clinically diagnosed as brain death, approximately 1,113 families were provided with information regarding organ donation, and 105 cases resulted in actual organ donations.^3^ To close the gap between the number of brain-dead patients and brain-dead organ donors, it is necessary to support and promote the development of systems in five-category hospitals and to build an environment that helps these hospitals to engage in organ donation. This effort will also contribute to fulfilling the wishes of citizens who hope to donate their organs after death.

According to the 2021 Cabinet Office opinion poll on citizens’ wishes regarding organ donation,^5^ 39.5% of respondents expressed a desire to donate their organs upon brain death or cardiac arrest. Furthermore, when an individual had previously indicated their willingness to donate, 90.9% of families reported that they wished to respect that decision. Respecting the wishes of the donor, their family, and transplant candidates is not only the responsibility of five-category hospitals but also a fundamental social duty of all medical institutions.

However, despite being classified as five-category hospitals, some have not well-established systems for brain-dead organ donation. The reasons cited include a lack of manpower and equipment, as well as deficiencies in operational manuals.^3^ Furthermore, the implementation of brain-dead organ donation significantly impacts routine medical practice,^6^ and medical staff who have experienced it firsthand report feeling burdened, highlighting the need for support.^7^ The burden associated with organ donation is immense in terms of human resources, time, finances, and psychological stress. Without proper system development, expanding the number of hospitals capable of organ donation cannot progress.^8^ Hence, developing an appropriate brain-dead organ donation system that also alleviates the burden on medical staff is of critical importance.

Therefore, this study investigated how five-category hospitals, which have conducted one or more brain-dead organ donations, have developed their donation systems. We examined specific components—such as discussions with the families of potential donors, as mandated in Guideline 6-1 (hereafter referred to as “organ donation option”)—and assessed how these elements influence donation numbers. By clarifying how to accurately capture patients’ wishes and support the full donation process, our findings aim to guide system developments that increase brain-dead organ donations and better serve transplant candidates in Japan nationwide.

## Methods

### Study design and participants

An anonymous self-administered web-based questionnaire survey was conducted by mail targeting the directors of 148 hospitals that had experienced at least one case of brain-dead organ donation between January 1, 2018, and December 31, 2023. These hospitals were publicly listed on the website of the Japan Organ Transplant Network as having performed brain-dead organ donations. The request letter specified that the respondent should be someone well-versed in the facility’s internal organ donation system (such as an in-hospital donor coordinator, if available). The survey requests and responses were sent and collected, respectively, between May 27 and August 31, 2024. The period addressed by the survey spanned from April 1, 2021, to March 31, 2024 (fiscal years, hereafter referred to as “FY”, 2021 to 2023).

### Measures

The following variables were assessed: Hospital characteristics; the number of patients with potential for organ donation (hereafter referred to as “potential donors”); the number of organ donation options (referring to instances in which medical professionals present the possibility of organ donation to the families of potential donors, in accordance with Guideline 6-1); the number of deceased organ donations (including brain-dead donation and donation after circulatory death; the count is by donor and not by organ); the number and professional roles of in-hospital donor coordinators (hereafter referred to as “IHCos”); and the status of in-hospital organ donation system development.

### Statistical analyses

Data processing and analysis were performed using IBM SPSS V.28.0, and p<0.05 was considered statistically significant. A simple aggregation was performed. The average numbers of organ donation options and diseased organ donations over three years were calculated, followed by correlation analysis. The normality of the numbers of organ donation options and organ donation from the deceased was tested using the Shapiro-Wilk test, confirming that the data were non-normally distributed. Consequently, comparisons of organ donation options, organ donations from the deceased, organ donation system development status, and the number and types of IHCos were conducted using the Mann-Whitney U-test.

### Ethical considerations

This study was conducted following a review by the Ethics Committee for Medical Research at Fujita Health University and was approved by the university president (HM24-025, approval date: May 21, 2024). There are no conflicts of interest to declare.

## Results

Responses were obtained from 84 out of 148 hospitals (response rate: 56.8%), and all responses were deemed valid (valid response rate: 100%).

### Sample profile (Table 1)

Among the hospitals, 28 (33.3%) had fewer than 500 beds, while 56 (66.7%) had 500 or more beds. There were 58 emergency and critical care centers (69.0%), 31 core/branch facilities of the Japan Neurosurgical Society (36.9%), 25 university hospitals (29.8%), 24 designated training facilities of the Japanese Association for Acute Medicine (28.6%), and 7 member hospitals of the Japanese Association of Children’s Hospital and Related Institutions (8.3%). A total of 52 hospitals (61.9%) were organ transplant centers.

The average annual number of emergency patient admissions per hospital was 5,520.6±3,229.9 cases (mean±SD, hereafter the same). In FY2023, the average number of deaths in departments where brain-dead organ donation could potentially occur was 153.7±151.9, the average number of patients expected to reach brain death was 8.5±9.1, the average number of potential donors was 6.0±7.8, and the average number of notifications to the relevant departments was 3.4±4.6 cases.

### Status of in-hospital organ donation system development (Table 2)

Regarding the development of organ donation systems, 81 hospitals (96.4%) had established in-hospital manuals for brain-dead organ donation, 72 hospitals (85.7%) had set up committee for brain-dead organ donation, and 57 hospitals (67.9%) conducted regular simulation of brain death diagnosis or organ donation process. Additionally, 45 hospitals (53.6%) held regular training sessions for organ transplantation, and 60 hospitals (71.4%) engaged in awareness campaigns for patients and families (posters, brochures, etc.). Furthermore, 30 hospitals (35.7%) had a system for confirming patient intent, such as questionnaires.

A total of 45 hospitals (53.6%) participated in the *Organ Donation Institution Collaboration Initiative* by Japan’s Ministry of Health, Labour and Welfare. Of these, 14 hospitals (31.1%) were designated as experienced core institutions, while 31 hospitals (68.9%) were inexperienced affiliate institutions.

Sixty-four hospitals (76.2%) had an in-hospital system for providing organ donation options. The healthcare professionals who provided these options included attending physicians in 62 hospitals (96.9%), IHCos in 20 hospitals (31.3%), and nurses in 3 hospitals (4.7%). The methods used included verbal communication alone in 39 hospitals (61.9%), brochures only in 9 hospitals (14.3%), and both methods in 15 hospitals (23.8%). Forty-one hospitals (48.8%) systematically detected potential donors.

### Status of IHCo positioning (Tables 3 and 4)

Among the respondent hospitals, 81 (96.4%) had IHCos, with an average of 7.0±6.0 per hospital. Of these, 16 hospitals (19.0%) had full-time IHCos.

By profession, nurses were the most commonly assigned as IHCos, present in 97.5%, with an average of 5.0±3.9 per hospital. Physicians were assigned in 49.4%, averaging 1.7±3.0. Administrative staff accounted for 26.6%, with 0.6±1.3 per hospital, while medical technicians were present in 24.1%, averaging 0.4±0.9 per hospital.

### Organ donation options, deceased organ donation cases, and in-hospital organ donation system development

#### 1) Organ donation options and deceased organ donation cases (Table 5)

The average number per hospital of organ donation options (one potential donor represents one option) presented by medical personnel (mean±SD/median) was 1.4±1.9/1.0 in FY2021, 1.8±2.7/1.0 in FY2022, and 2.1±2.9/1.0 in FY2023. The average over the three-year period was 1.9±2.5/0.7 cases.

The number of deceased organ donation cases (one donor represents one case) was 0.6±0.8/0.0 cases in FY2021, 0.8±1.3/0.0 cases in FY2022, and 0.8±1.4/0.0 cases in FY2023. The three-year average was 0.7±1.0/0.3.

#### 2) Correlation between organ donation options and deceased organ donation cases over three years (Figure 1)

A positive correlation was observed between the number of organ donation options and the number of deceased organ donation cases over the three-year period (r=0.484, p<0.001).

#### 3) Comparison based on in-hospital organ donation system development, number and type of IHCos (Table 6)

The number of organ donation options was significantly higher in hospitals with six or more IHCos (p=0.003) and in those that systematically detected potential donors (p=0.022). Moreover, the number of deceased organ donation cases was significantly higher in hospitals with full-time IHCos (p=0.031).

## Discussion

This study led to three main findings:

1) Ninety-six percent of hospitals with experience in brain-dead organ donation had established in-hospital manuals and appointed IHCos, which included nurses, physicians, and administrative staff.

2) Hospitals with six or more IHCos, or that routinely screened for potential donors, presented significantly more organ donation options.

3) The number of organ donation options presented showed a significant positive correlation with the number of deceased organ donation cases.

Based on these findings, five-category hospitals should consider optimizing their organ donation systems to accurately capture patients’ wishes and ensure the successful completion of the donation process.

### In-hospital organ donation system development and the Organ Donation Institution Collaboration Initiative

In FY2023, hospitals saw an average of 6.0 potential donors but issued only 3.4 notifications to the relevant departments. While some of this gap may be due to families choosing not to proceed, it may also reflect insufficient internal procedures for contacting the appropriate departments. To address this, integrating organ donation considerations into routine clinical workflows is essential.

Some five-category hospitals still default to donation after cardiac arrest because brain-death pathways are not fully established.^9^ However, when a patient has expressed a desire for brain-dead organ donation and the family wishes to respect their intention, it is a fundamental responsibility of hospitals to respect and fulfill that wish. Therefore, advancing the development of systems for brain-dead organ donation is essential. This study has identified specific measures that can help to achieve this goal.

Nearly 90% of respondent hospitals had established in-hospital manuals for brain-dead organ donation and committee for brain-dead organ donation. However, fewer than 70% conducted regular simulations involving brain death determination and organ donation, and only about 50% offered regular training sessions for organ transplantation. As a result, opportunities for healthcare professionals to acquire relevant knowledge and skills remain limited, undermining preparedness for the rare occurrence of brain-dead organ donation—on average fewer than one case per year. Therefore, besides improving the system, optimizing education and training programs for healthcare professionals is also essential.

Especially for hospitals with a low number of organ donations, the *Organ Donation Institution Collaboration Initiative* provides a valuable resource. The number of core hospitals with extensive experience in brain-dead organ donation is expected to reach 31 nationwide in FY2025, increasing from 17 and 25 hospitals in FY2023 and FY2024, respectively,^10^ while affiliate hospitals increased to 206 in FY2024.^4^ By sharing in-hospital system, development methods from experienced core hospitals with other five-category hospitals, even those with less experience can gain clarity on how to proceed.

The Ministry of Health, Labour and Welfare^11^ has reported the effects of the *Organ Donation Institution Collaboration Initiative*, highlighting an increase in the proportion of brain-dead organ donations from hospitals participating in the initiative. Both the number of core hospitals and affiliate hospitals involved in the initiative have shown an upward trend. In FY2022, 50% of brain-dead organ donations came from hospitals participating in the initiative. From this, it can be concluded that participation in the initiative contributes to promoting in-hospital system development and, as an outcome, leads to an increase in the number of organ donations.

### The role of in-hospital donor coordinators (IHCos)

#### 1) Number and full-time allocation

Hospitals averaged 7.0±6.0 IHCos, and 19.0% employed at least one full-time IHCo. Hospitals with six or more IHCos offered more organ donation options, and those with a full-time IHCo conducted more organ donations from the deceased. This demonstrates that adequate IHCo staffing promotes both option presentation and actual donation.

Having a larger number of IHCos suggests that a hospital is proactive in developing an organ donation system and securing cooperation across departments. For example, one report described how expanding an IHCo team from three nurses to a multidisciplinary group of ten—combined with regular meetings—enhanced coordination and responsiveness, ultimately increasing the number of donations.^12^ Another hospital reported that distributing roles among its 18 IHCos, including attending physicians and ward nurses, improved efficiency in supporting families during decision-making.^13^ Thus, a large, multidisciplinary IHCo team can help enable efficient donor identification and donation processes, even in hospitals where organ donations occur infrequently.

#### 2) Professional composition among IHCos

Nurses served as IHCos in 98% of the respondent hospitals, physicians in 50%, administrative staff or medical technologists in approximately 25%, aligning with previous findings in Japan.^14^ While the professional composition of IHCos may differ depending on the hospital, a standard structure is recommended based on this study.

Nurses play a key role in organ donation due to their specialized skills and experience, making them ideally suited for the IHCo role. They frequently interact with patients and their families, fostering trust—a key factor when discussing delicate topics such as organ donation. Their proximity to patients allows them to support donor families in decision-making and provide psychological care, facilitating the consent process. In the context of end-of-life care, nurses frequently provide support to patients and their families in coping with psychological distress and grief. Given their close involvement in these situations, they are well-positioned to play a critical role in facilitating family decision-making processes. Additionally, nurses collaborate daily with multiple professions in clinical practice, which helps them develop strong coordination and communication skills.

Hospitals with extensive experience in organ donation have increasingly adopted multidisciplinary IHCo teams, including physicians, ward nurses, operation room nurses, medical social workers (MSW), clinical psychologists, medical technicians, and administrative staff. By establishing such teams, responsibilities are distributed more efficiently, reducing the burden on any one individual.^15^ Since diseased organ donation is infrequent and unpredictable, continued in-hospital system development from a multidisciplinary collaboration perspective is essential.

### Systematic detection of potential donors

Only about half of the respondent hospitals systematically identified potential donors, yet those that did recorded significantly more organ donation options and actual organ donations. Identifying potential donors enables the presentation of options, which can help uncover patients’ previously unexpressed willingness to donate, ultimately leading to actual donations. Systematizing the identification process therefore increases the number of actual donations.

The low number of organ donations in Japan is not primarily due to a lack of willing donors, but rather to challenges in identifying potential donors and connecting them to the organ donation process.^16^ Thus, improving the identification of potential donors should be a logical starting point for enhancing organ donation rates. This conclusion is supported by studies from countries in which high organ donation rates were achieved.^17–19^

Identifying potential donors is a responsibility of IHCos affiliated with departments where brain-dead organ donation may occur, as they are closest to the patients. However, without a structured hospital-wide approach, these efforts risk becoming overly individualized and may lead to misunderstandings or negative perceptions—such as being labeled as “organ harvesting”.^20^ Therefore, it is essential to establish a well-developed in-hospital system and ensure that IHCos receive consistent understanding and support from other staff members.

### Organ donation option presentation and donation outcomes

#### 1) Presentation methods

Among the respondent hospitals, 62% relied on verbal communication alone, 14% provided only brochures, and 24% used both. Attending physicians delivered the presentation in 97% of cases.

Although brochures reduce physicians’ psychological stress,^21^ written information alone is not sufficient, and a direct dialogue between the patient’s family and healthcare professionals remains essential.^22^ In this study, medical professionals verbally presented the option in two-thirds of hospitals. For hospitals unfamiliar with the option presentation process, combining brochures with verbal communication may lower the barrier to initiating the conversation. In 31% of hospitals, IHCos also presented options, usually in collaboration with attending physicians and nurses to share information, while adjusting the timing of the presentation to align with the family’s circumstances.

#### 2) Correlation between organ donation options and actual organ donations

A significant positive correlation was observed between the number of organ donation options presented and the number of organ donations from the deceased. In brain-dead organ donations under the revised Organ Transplant Act, provision of organ donation information was identified as the trigger in 68.5% of cases.^1^ Given this, an increase in option presentations naturally should lead to a rise in actual organ donations.

To efficiently increase the number of options, an integrated and systematic approach is necessary, including: well-developed donation systems, adequate IHCo staffing, and systematic detection of potential donors. For hospitals that lack information on specific measures, participating in the *Organ Donation Institution Collaboration Initiative* and receiving guidance from core hospitals would be highly beneficial. Accurate donor identification, incorporation of the donation option into end-of-life care, and prepared manuals for brain-death determination and organ retrieval are all crucial for five-category hospitals to fulfil their social responsibilities.

Expanding the number of hospitals that can provide optimal care and properly address requests from patients and their families will strengthen regional donation systems and advance Japan’s nationwide framework for brain-dead organ donation.

### Limitations of the Study

This study focused exclusively on hospitals that were publicly identified as having conducted brain-dead organ donations, and the response rate was below 60%. While this presents certain limitations, the study holds significance as the first to provide a detailed analysis of the organ donation systems in five-category hospitals in Japan. Moving forward, further qualitative investigations are necessary to deepen the understanding of the ideal approach to in-hospital organ donation system development.

## Conclusion

Almost all respondent hospitals with experience in brain-dead organ donation had appointed IHCos. A significant positive correlation was observed between the number of organ donation options presented and the actual number of organ donations. Hospitals with six or more IHCos, or a system for systematically detecting potential donors, had more organ donation options, while those with dedicated full-time IHCos had significantly more diseased organ donations. IHCos contribute to the accurate identification of potential donors and early intervention. Establishing an appropriate number of IHCos and developing a system for detecting potential donors are essential for properly capturing the intentions of patients and their families and ensuring the completion of the organ donation process.

## Figures and Tables

**Figure 1:**
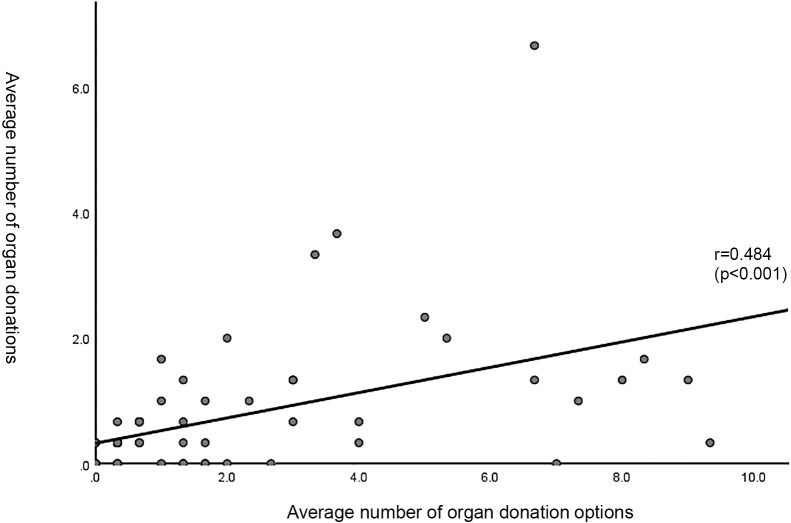
Correlation between organ donation options and organ donations (three-year averages). A positive correlation was observed between the numbers of organ donation options and organ donations (r=0.484, p<0.001). The dots represent respondent hospitals.

**Table 1:** Characteristics of the respondent hospitals (n=84) Mean±SD [range]

		n	%
Region	Hokkaido	1	1.2%
	Tohoku	2	2.4%
	Kanto-Koshinetsu	24	28.6%
	Tokai-Hokuriku	26	31.0%
	Kinki	10	11.9%
	Chugoku-Shikoku	8	9.5%
	Kyushu-Okinawa	13	15.5%
Bed number	<500	28	33.3%
	500=<	56	66.7%
Five category hospitals (multiple responses)	Emergency and critical care center	58	69.0%
	Core/branch facility of the Japan Neurosurgical Society	31	36.9%
	University hospital	25	29.8%
	Designated training facility of the Japanese Association for Acute Medicine	24	28.6%
	Member hospital of the Japanese Association of Children’s Hospitals and Related Institutions	7	8.3%
Organ transplant center	Yes	52	61.9%
	No	32	38.1%
Average annual number per hospital of emergency patient accepted (n=80 excluding no responses)		5,520.6±3,229.9 [500–15,922]	
FY 2023 average number of deaths at units with potential organ donors (n=60 excluding no responses)		153.7±151.9 [0–878]	
FY 2023 average number of brain-dead patients (n=62 excluding no responses)		8.5±9.1 [0–50]	
FY 2023 average number of potential organ donors (n=65 excluding no responses)		6.0±7.8 [0–37]	
FY 2023 average number of notifications to the relevant departments (n=59 excluding no responses)		3.4±4.6 [0–19]	

※Due to rounding the numbers, the total may differ slightly from 100%.

**Table 2:** Status of in-hospital organ donation system development (84 respondent hospitals)

		n	%
Activities (multiple responses)	In-hospital manuals for brain-dead organ donation	81	96.4%
	Committee for brain-dead organ donation	72	85.7%
	Regular simulation of brain death diagnosis or organ donation process	57	67.9%
	Regular training session for organ transplantation	45	53.6%
	Awareness campaign for patient and family (poster, brochure, etc.)	60	71.4%
	Patient intent confirmation system (using questionnaires, etc.)	30	35.7%
	Others	1	1.2%
Organ Donation Institution Collaboration Initiative	Joining	45	53.6%
	Not joining	39	46.4%
If the response was ‘Joining’ Facility category	Experienced core institution	14	31.1%
	Inexperienced affiliate institution	31	68.9%
In-hospital system for providing potential donation options	Present	64	76.2%
	Not present	20	23.8%
If the response was ‘Present’			
Health care professionals of providing organ donation options (multiple responses)	Attending physician	62	96.9%
	In-hospital donor coordinator	20	31.3%
	Primary nurse	3	4.7%
	Others	2	3.1%
Methods of presenting options	Verbal communication alone	39	61.9%
	Providing only brochures	9	14.3%
	Both	15	23.8%
Activity for systematic potential donor detection	Active	41	48.8%
	Not active	43	51.2%

**Table 3:** In-hospital donor coordinators (84 respondent hospitals)

		n	%	Mean±SD [range]
In-hospital donor coordinator (s)	Present	81	96.4%	
	Not present	3	3.6%	
Total number of in-hospital donor coordinators per facility				7.0±6.0 [1–36]
Full-time in-hospital donor coordinator(s)	Present	16	19.0%	
	Not present	65	77.4%	

**Table 4:** Job category representation and their average number among IHCos (79 respondent hospitals excluding no responses)

	Present (% of hospitals with IHCos from this category) %	Average number as IHCos per hospital Mean±SD [range]
Nurse	97.5%	5.0±3.9 [0–23]
Physician	49.4%	1.7±3.0 [0–9]
Administrative staff	26.6%	0.6±1.3 [0–8]
Medical technologist	24.1%	0.4±0.9 [0–4]
Others	31.6%	0.4±0.8 [0–4]

**Table 5:** Number of organ donation options and organ donations from the deceased

		Average per hospital±SD	[range]	Median
Number of organ donation options	FY 2021 (n=68)	1.4±1.9	[0–7]	1.0
	FY 2022 (n=68)	1.8±2.7	[0–12]	1.0
	FY 2023 (n=71)	2.1±2.9	[0–12]	1.0
	Three-year average	1.9±2.5	[0–9.3]	0.7
Number of organ donations from the deceased (including brain-dead donation and donation after circulatory death)	FY 2021 (n=78)	0.6±0.8	[0–4]	0.0
	FY 2022 (n=81)	0.8±1.3	[0–6]	0.0
	FY 2023 (n=80)	0.8±1.4	[0–10]	0.0
	Three-year average	0.7±1.0	[0–6.7]	0.3

**Table 6:** Comparison based on the status of the in-hospital system for organ donation

		Number of organ donation options			Number of organ donations from the deceased (including brain-dead donation and donation after circulatory death)		
		n	median	p	n	median	p
Joining Organ Donation Institution Collaboration Initiative	Yes	37	0.7	NS	43	0.3	NS
	No	34	0.7		38	0.3	
Having in-hospital donor coordinators	Yes	68	0.7	NS	78	0.3	NS
	No	3	0.0		3	0.0	
Number of in-hospital donor coordinators	up to five	39	0.3	0.003	45	0.3	NS
	six and more	29	1.7		33	0.3	
Having full-time in-hospital donor coordinators	Yes	13	0.7	NS	15	1.3	0.031
	No	55	0.7		63	0.3	
Systematic detection of potential organ donors	Yes	34	1.3	0.022	39	0.3	NS
	No	37	0.3		42	0.3	
In-house organ donation option system	Yes	55	0.7	NS	61	0.3	NS
	No	16	1.0		20	0.3	

Mann-Whitney’s U test n: excluding no responses

## Supplementary Material

PDF-Japanese
