# Supplementary material for: In-hospital systems for organ donation after brain death in Japan: : A nation-wide survey—advancing brain-dead organ donation systems in Japan—
Source: Fujita Med J. 2025 Nov 5;12(1):33–9. doi: 10.20407/fmj.2025-007 (PMC12865278; doi:10.20407/fmj.2025-007)
Supplement: Supplementary file 1 — PDF-Japanese [file fmj-12-033-s001.pdf]

■ 原 著

脳死下臓器提供における施設内体制の現状と課題
－日本の臓器提供施設における実態調査－

高木友貴, 朝居朋子, 田﨑あゆみ, 中村小百合
藤田医科大学大学院保健学研究科

In-Hospital Systems for Organ Donation after Brain Death in Japan: A Nation-wide Survey
- Advancing Brain-dead Organ Donation Systems in Japan -

要旨：本邦の脳死下臓器提供件数は､諸外国と比べて著しく少なく､臓器提供施設の体制整備が不十分である｡本研究は、脳死下臓器提供経験施設の体制整備状況を調査し､施設内体制整備の望ましい在り方について検討することを目的とした｡2018～2023 年に脳死下臓器提供を 1 件以上経験した 148 施設のうち、協力の得られた 84 施設に対しオンラインによる無記名自記式調査を実施した(回収率 57%)｡96%の施設に院内ドナーコーディネーター(院内 Co)が設置されており、1 施設あたり 7.0±6.0 人、院内 Co の職種は看護師 98%・医師 50%・事務職員 27%であった。臓器提供選択肢提示件数と臓器提供件数には有意な正の相関があった。院内 Co が 6 人以上又はポテンシャルドナーを把握する体制があると選択肢提示件数が多く、専従・専任院内 Co がいると脳死下及び心停止後臓器提供件数が有意に多かった。院内 Co の適正設置とポテンシャルドナー把握体制の構築は、患者の臓器提供の意思を適切に汲み取り､臓器提供を完遂する体制整備に資すると考える。

キーワード
脳死下臓器提供, 施設内体制整備, 院内ドナーコーディネーター, 臓器提供選択肢提示

1．はじめに

日本において､脳死下臓器提供は 1997 年の臓器移植法施行によってはじめて可能となった｡同法下では本人の書面による意思表示が必須であったが、2010 年の改正臓器移植法施行により、本人の意思が不明の場合（拒否の意思表示がない場合に限る）家族の承諾のみで脳死下臓器提供が可能となった｡その結果、日本の脳死下臓器提供件数は改正法施行前 86 件､施行後 1,064 件、うち家族承諾 843 件(79.2%)(2024 年 12 月 31 日現在)[1] となり､脳死下臓器提供件数が増加している｡しかし、移植希望登録者数も年々増加しており、現在約 16,000 人の登録者に対し、1 年間に移植を受けられたのは約 600 人、4%程度である [2]。

臓器移植法ガイドライン第 4 の 3 で、脳死下臓器提供ができる施設は高度な救急医療が施せる 5 類型施設（大学附属病院､日本救急医学会の指導医指定施設､日本脳神経外科学会の基幹施設又は連携施設､救命救急センターとして認定された施設､日本小児総合医療施設協議会の会員施設）と定められている。しかし、現状では臓器提供施設の体制整備は全ての 5 類型施設でなされていない。厚生労働省 [3] によると､5 類型施設は全国に 906 施設あるが、465 施設(51%)は体制が整っておらず(2024 年 3 月末現在)、これまでに脳死下臓器提供の経験のある施設は 297 施設、そのうち経験件数 1 例が 110 施設(37.0%)で最多であり､10 例以上が 17 施設(5.7%)しかない。この現状に対し、厚生労働省は、臓器提供施設が脳死判定や終末期対応に不慣れで、脳死下臓器提供の可能性がある患者に脳死の判断が行われておらず、また 家族への臓器提供の情報提供ができていない可能性がある

と分析している[4]。従って、臓器提供施設の施設内体制整備は急務である。

さらに、脳死者 4,412 人(推計)、脳死とされうる状態と診断された者 1,363 人(推計)、家族に臓器提供に関する情報が提供された者 1,113 人(推計)、脳死下臓器提供に至った者 105 人(実数)である[3]。脳死者数と脳死下臓器提供件数のギャップを解決するには、5 類型施設の体制整備を支援・促進し、施設が臓器提供に取り組める体制を構築する必要がある。それは、国民の臓器提供の希望の成就を支援することにもつながる。

国民の臓器提供の希望に対する内閣府世論調査(2021 年)[5]によると、脳死下または心停止下の臓器提供を希望する者は 39.5%、本人が提供意思を表示していた場合、家族が本人の意思を尊重することを希望するのは 90.9%である。本人の意思と家族の希望を尊重し、臓器移植を希望する患者につなげるのは 5 類型施設の他すべての医療機関の社会的責務と言っても過言ではない。

しかしながら、5 類型施設でありながら脳死下臓器提供体制整備をしない理由として、マンパワーや設備の不足、マニュアル不備等が挙げられている[3]。また、脳死下臓器提供の実施は日常診療への影響が大きく[6]、実際に経験した医療スタッフが脳死下臓器提供に負担感を抱いており、支援の必要性が指摘されている[7]。臓器提供に係る負担は人的・時間的・経済的および精神的に膨大であるため、体制整備が不十分なままでは提供施設の拡大を進めることはできない[8]。従って、施設として脳死下臓器提供に関わる医療スタッフの負担を軽減し、支援する体制を整えること、即ち適切な体制整備をすることは非常に重要である。

そこで、本研究では脳死下臓器提供を経験した 5 類型施設の施設内体制整備の実態、施設内体制整備の具体的内容が選択肢提示（臓器移植法ガイドライン第６の１「臓器提供の可能性がある患者の家族に臓器提供の機会があることを告げること」を指す）及び臓器提供の件数に与える影響を明らかにした。これにより患者の臓器提供の意思を適切に汲み取り、臓器提供を完遂する体制を整えるためにすべきことが明らかになり、臓器提供体制整備の課題解決、ひいては脳死下臓器提供件数の増加と移植希望登録者数が期待を持てるような社会の実現に寄与できると考える。

２．研究の方法

１）研究対象

2018 年 1 月 1 日～2023 年 12 月 31 日の 6 年間に、脳死下臓器提供を 1 件以上経験し、公益社団法人日本臓器移植ネットワークホームページに脳死臓器提供事例を行なったとして施設名が公表されている 148 施設の施設長に対して、郵送で調査を依頼した。依頼文には、回答者は「臓器提供に関する施設内体制を熟知している者(いる場合は院内 Co 等)」と記載した。

２）データの収集方法

無記名自記式 WEB アンケート調査。

３）調査期間

調査の依頼・回収は 2024 年 5 月 27 日～2024 年 8 月 31 日。調査内容の対象期間は 2021 年 4 月 1 日～2024 年 3 月 31 日（2021 年度～2023 年度）。

４）調査内容

回答施設の属性、臓器提供の可能性のある患者（以下、ポテンシャルドナー）数、選択肢提示件数、脳死下及び心停止後臓器提供件数、院内ドナーコーディネーター（以下、院内 Co）の人数と職種、臓器提供体制整備状況。

５）データの分析方法

単純集計を行なった。選択肢提示件数と脳死下及び心停止後臓器提供件数は 3 年間の平均値を出し、相関分析を行なった。選択肢提示件数と脳死下及び心停止後臓器提供件数は、正規性の検定（Shapiro-Wilk test）により非正規分布であることを確認した。そのため、選択肢提示件数、脳死下及び心停止後臓器提供件数、臓器提供体制整備状況、院内 Co の人数及び種別による比較には Mann-Whitney’s U-test を用い、$p<0.05$ をもって有意差ありと判断した。統計解析には IBM SPSS Statistics ver.28 を用いた。

６）倫理的配慮

本研究の実施に際しては、藤田医科大学医学研究倫理審査委員会の審査を経て、学長の承認を得た(HM24-025、承認年月日:2024 年 5 月 21 日)。申告すべき利益相反はない。

３．結果

148 施設中 84 施設から回答が得られ(回収率:56.8%)、全て有効回答とした(有効回答率 100%)。

１）回答施設の属性（表 1）

病床数は 500 床未満 28 施設(33.3%)、500 床以上 56 施設(66.7%)、救急救命センター58 施設(69.0%)、日本脳神経外科学会の基幹施設または連携施設 31 施設(36.9%)、大学附属病院 25 施設(29.8%)、日本救急医学会の指導医指定施設 24 施設(28.6%)、日本小児総合医療施設協議会の会員施設 7 施設(8.3%)であった(複数回答)。52 施設(61.9%)が臓器移植施設であった。

年間救急搬送受入件数(平均±SD、以下同じ)5,520.6±3,229.9 件、2023 年度の脳死下臓器提供発生可能部署における死亡者数 153.7±151.9 人、脳死が見込まれた患者数 8.5±9.1 人、ポテンシャルドナー数 6.0±7.8 人、関連部署への連絡件数 3.4±4.6 件であった。

２）臓器提供体制整備状況（表 2）

臓器提供体制整備の内容としては、施設内マニュアル整備 81 施設(96.4%)、臓器提供関連委員会の設置 72 施設(85.7%)、脳死判定・臓器提供シミュレーション定期開催 57 施設(67.9%)、移植医療研修会の定期開催 45 施設(53.6%)、患者家族への普及啓発(ポスター掲示やパンフレット配布等)60 施設(71.4%)、患者意思確認システム(問診票等による意思確認システム等)30 施設(35.7%)であった。

臓器提供施設連携体制構築事業参加施設 45 施設(53.6%)、施設区分は拠点施設 14 施設(31.1%)、連携施設 31 施設(68.9%)であった。

ポテンシャルドナーへの選択肢提示実施体制があるのは 64 施設(76.2%)、実施者は担当医 62 施設(96.9%)、院内 Co20 施設(31.3%)、担当看護師 3 施設(4.7%)、方法は口頭のみ 39 施設(61.9%)、パンフレットのみ 9 施設(14.3%)、口頭とパンフレットの併用 15 施設(23.8%)であった。ポテンシャルドナーを定期的に把握しているのは 41 施設(48.8%)であった。

３）院内 Co の設置状況（表 3,4）

院内 Co 設置施設は 81 施設(96.4%)、1 施設当たり 7.0±6.0 人(平均±SD、以下同じ)、専任・専従の院内 Co がいるのは 16 施設(19.0%)であった。

院内 Co の職種別でみると、最多は看護師で 97.5%の施設に設置されており 1 施設あたり 5.0±3.9 人、次いで医師 49.4%・1.7±3.0 人、事務職員 26.6%・0.6±1.3 人、臨床検査技師 24.1%・0.4±0.9 人であった。

４）選択肢提示件数及び脳死下及び心停止後臓器提供件数と臓器提供体制整備

①選択肢提示件数及び脳死下及び心停止後臓器提供件数（表 5）

医療者からの選択肢提示件数(平均±SD/中央値、以下同じ)は、2021 年度 1.4±1.9/1.0 件、2022 年度 1.8±2.7/1.0 件、2023 年度 2.1±2.9/1.0 件、3 年間では 1.9±2.5/0.7 件であった。

脳死下及び心停止後臓器提供件数は、2021 年度 0.6±0.8/0.0 件、2022 年度 0.8±1.3/0.0 件、2023 年度 0.8±1.4/0.0 件、3 年間では 0.7±1.0/0.3 件であった。

②3 年間の選択肢提示件数と脳死下及び心停止後臓器提供件数の相関(図 1)

3 年間の選択肢提示件数と脳死下及び心停止後臓器提供件数には正の相関があった(r=0.484、$p<0.001$)。

③臓器提供体制整備状況や院内 Co の人数・種別による比較（表 6）

院内 Co の人数が 6 人以上の場合(p=0.003)、ポテンシャルドナーを定期的に把握する活動をしている場合(p=0.022)は、選択肢提示件数が有意に多かった。専従・専任院内 Co がいる場合、脳死下及び心停止後臓器提供件数が有意に多かった(p=0.031)。

４．考察

本研究で明らかになったことは、①脳死下臓器提供の経験のある施設の 96%で施設内マニュアル整備や院内 Co 設置（看護師・医師・事務職員等）がなされていたこと、②院内 Co が 6 人以上及びポテンシャルドナーを定期的に把握する活動をしている施設では選択肢提示件数が有意に多いこと、③選択肢提示件数と脳死下及び心停止後臓器提供件数には有意な正の相関があったことであった。そこで、5 類型施設として、患者の臓器提供の意思を適切に汲み取り、臓器提供を完

遂する体制を整えるための臓器提供体制整備の望ましい在り方(リコメンデーション)について考察する。

１）臓器提供体制整備の在り方と臓器提供施設連携体制構築事業の意義

2023 年度は、ポテンシャルドナー数(平均)6.0 件、関連部署への連絡件数(平均)3.4 件であった。ポテンシャルドナーの存在が関連部署に連絡されない理由としては、家族が臓器提供を望まないこと以外に、連絡先が施設内に把握されていないことも考えられる。即ち、臓器提供に向けてのプロセスが一部途切れている可能性があると思われる。この可能性をできるだけ無くすために、臓器提供が日常業務となり得るような体制整備をする必要がある。

また、5 類型施設であっても、脳死下臓器提供の体制が未整備な場合、結果として脳死下臓器提供が叶わず、心停止下臓器提供に至っている症例がある[9]。本人に脳死下臓器提供の意思があり、家族もその意思の尊重を希望する場合、それを実現することは医療機関に課せられた責務である。そのためには、脳死下臓器提供の体制整備を進めねばならないが、具体的にどうしたらいいかを今回の研究で明らかにした。

回答施設の 9 割近くで、施設内マニュアル整備、臓器提供関連委員会の設置がなされていたが、脳死判定・臓器提供シミュレーション定期開催は 7 割未満、移植医療研修会の定期開催は約 5 割で、医療者を対象にした知識や技術を習得する場の設定はより少ないことが判明した。臓器提供は年 1 件にも満たない稀少な事例であるからこそ、災害対策と同じで日常的にいかに備えるかが欠かせない。そのためには、施設内で体制整備をしつつ、医療者に対する教育研修も施す必要がある。

しかし、臓器提供件数の少ない施設にとっては何をどうしたらいいかが難しい。そこに臓器提供施設連携体制構築事業の意義がある。脳死下臓器提供の経験が豊富な拠点施設（令和 7（2025）年度）は全国 31 施設となり、令和 5(2023)年度 17 施設、令和 6(2024)年度 25 施設と年々増加している[10]。連携施設も令和 6(2024)年度 206 施設になった[4]。経験が豊富な拠点施設がその体制整備の方法を他施設に提供することで、経験の少ない施設でもどのように進めていけばいいかが明白となり、施設内体制整備の基盤作りとなると考えられる。それにより、5 類型施設の体制整備が促進されることが期待できる。

厚生労働省[11]は、臓器提供施設連携体制構築事業の効果として、脳死下臓器提供事例のうち、臓器提供施設連携体制構築事業に参加している施設からの提供の割合が増加していること、拠点施設及び連携施設への参加数はいずれも増加傾向にあり、令和 4(2022)年度の脳死下臓器提供の 50％が事業に参画している施設からの提供事例であったことを報告している。このことから、臓器提供施設連携体制構築事業への参画は、施設内体制整備の促進とそのアウトカムとしての臓器提供件数の増加につながると言える。

２）院内 Co の望ましい在り方

①院内 Co の適正人数と専従･専任院内 Co の配置

院内 Co の設置人数(平均)は 7.0±6.0 人、専従・専任者は 19.0％の施設にいた。院内 Co が 6 人以上いる施設は選択肢提示件数が有意に多く、専従・専任院内 Co がいる場合、脳死下及び心停止後臓器提供件数が有意に多かった。即ち、院内 Co の存在が選択肢提示件数や臓器提供件数の増加に直接影響を与えることが明らかになった。

院内 Co 数が多いということは、当該施設が臓器提供体制整備に対して前向きであり、多人数の院内 Co 配置に価値を見出し、各部署の協力を得ていると考える。院内 Co が看護師 3 人から多職種 10 人に増え、定例会を開催したことで、院内 Co 間の連携及び対応力が強化され、提供件数の増加につながったこと[12]が報告されている。また、18 人の院内 Co が居り、家族の意思決定支援には病棟医師・看護師が関わる等役割分担をすることで効率化を図っている施設もある[13]。従って、多人数・多職種の院内 Co の存在は、年数回と頻度の低い臓器提供事例であっても、継続的かつ効果的な活動が維持でき、さらに専従･専任院内 Co が居れば専門的に関わることができることを示唆している。

②院内 Co の構成

看護師の院内 Co は 98％の施設に居り、ほぼ全

ての施設で設置されていた。医師は1/2の施設で、事務職員と臨床検査技師は1/4の施設で設置されていた。院内Coの職種をどうするかは施設の意向に影響するが、本研究より一定の構成が推奨できたと言える。

特に看護師が多数を占め、中心的な役割を果たしている背景には、看護師特有のスキルや経験が臓器提供全体において重要であることが挙げられる。看護師は日常的に患者や家族と接する機会が多く、信頼関係を築く役割を担っており、この信頼関係は臓器提供に関するデリケートな話題を取り扱う際に極めて重要である。看護師が家族の意思決定を支援し、心理的なケアや感情的なサポートを提供することで、臓器提供の同意を得るプロセスを支える役割を果たすことからも、看護師の院内Coは最適であると言える。看護師は終末期医療において、患者や家族が直面する心理的負担や悲嘆に対してケアを提供する場面も多いため、家族の意思決定支援にも力を発揮することができる。看護師は多職種と協働して日常業務を遂行する中で、連携や調整能力を発展させている。この多職種連携の能力と経験は、家族、医療チーム、関連機関との情報共有や連携を円滑に進める役割を果たしている。特に、患者の状態を把握しながら、家族に寄り添い臓器提供の可能性を伝える調整役としての役割が求められている。

前述のように、院内Coとしての看護師は、臓器提供プロセス全体を調整し、スムーズな進行を可能にする中心的存在となる。患者に近い看護師が院内Coとして活動することは理にかなっているとされている[14]ことから、看護師を院内Coにすることは、臓器提供体制整備の在り方として望ましいと考える。また、本研究では、院内Coの職種としては看護師が最多で、次いで医師、事務職員、臨床検査技師であった。これは日本国内の現状を調査した先行研究と矛盾しない[14]。さらに、臓器提供経験の多い施設では複数かつ多職種の院内Coチームを構築する施設が増加し、医師を中心として病棟看護師、手術部看護師、MSW、臨床心理士、臨床検査技師、さらには事務職員が院内Coになることで、業務分担を行い、1人の負担を減らしている[15]。脳死下及び心停止後臓器提供は、件数は少ないものの、いつ生じるかわからないため、日常的に多職種連携の観点で体制整備に注力することが肝要である。

３）ポテンシャルドナーを定期的に把握する活動

本研究では、ポテンシャルドナーを定期的に把握している施設は約半数であったものの、ポテンシャルドナーを定期的に把握する活動をしていると選択肢提示件数が有意に多く、選択肢提示件数と臓器提供件数に正の相関があったことが明らかになった。即ち、ポテンシャルドナーを把握すれば、選択肢提示をすることができ、選択肢提示をすれば臓器提供の潜在的意思を救い上げ、臓器提供に至ることができる。従って、ポテンシャルドナーの把握と選択肢提示のルーチン化が臓器提供件数の増加を導くと言える。

日本で臓器提供件数が少ないのはドナーがいないからではなく、潜在的ドナーを臓器提供に結びつけることができていないからである[16]。即ち、ポテンシャルドナーの把握が臓器提供への始点である。このことは臓器提供件数の多い諸外国でも同様で、ポテンシャルドナーの把握で臓器提供件数が増加することが報告されている[17-19]。

ポテンシャルドナーを把握する活動は、その患者に最も近い脳死下臓器提供発生可能部署所属の院内Coに期待される業務である。しかし、施設として行う姿勢がないと「臓器狩り」[20]という誤解や否定的な印象を与える可能性がある。だからこそ、施設内体制を整備し、脳死下臓器提供発生可能部署所属の院内Coが日常的に他のスタッフの理解と協力を得る努力が求められる。

４）選択肢提示件数と脳死下及び心停止後臓器提供件数

①選択肢提示の方法

選択肢提示の方法は、口頭のみは62%、パンフレットのみは14%、口頭とパンフレットの併用は24%で、行うのは担当医が97%であった。

パンフレットのみであれば、家族からの返事を要求しないため、医師の負担の軽減となる[21]が、書面情報だけでは不十分で、患者家族と医療従事者の対話が患者にとって最適な選択を導く上で不可欠である[22]とされている。本研究の回答施設のうち、2/3の施設は口頭で行っていたが、選択肢

提示に慣れていない施設にとっては、パンフレットを併用することも選択肢提示へのハードルを下げることにつながるのではないかと考える。また、施設によっては、担当医の負担軽減のため、院内 Co(31%)が行っているが、その際も担当医や担当看護師と情報共有及び連携し、患者家族の状況に合わせて、適切なタイミングで行っているのではないかと考える。

②選択肢提示件数と脳死下及び心停止後臓器提供件数の関係性

本研究では、選択肢提示件数と脳死下及び心停止後臓器提供件数に有意な正の相関があった。改正臓器移植法後の脳死下臓器提供において、きっかけとして臓器提供に関する情報提供(選択肢提示)が 68.5%である [1] ことから、選択肢提示件数が増加することで臓器提供件数が増加することは必然である。

選択肢提示件数を増加させるためには、臓器提供体制整備、院内 Co 設置、ポテンシャルドナーを定期的に把握する活動等の組織的かつ融合的な体制及び活動が不可欠である。具体的に何をしたらいいか情報がない施設においては、臓器提供施設連携体制構築事業に参加し、拠点施設のアドバイスを受けるのは有意義である。ポテンシャルドナーを確実に把握し、適切に終末期医療の一環として選択肢提示を実施し、脳死判定や臓器摘出時の支援等ができるような人員配置やマニュアル作成をすることは、5 類型施設としての社会的責務を果たすことにつながる。そのような施設で患者が最善の医療を受けられ、臓器提供の希望があれば患者家族に適切な対応ができる施設が増えることは、地域における臓器提供体制の構築、ひいては日本全体の臓器提供体制の構築につながるはずである。

研究限界

本研究の調査対象は、脳死臓器提供事例を行なったとして施設名が公表されている施設に限定されており、かつ回収率も 6 割に満たなかった。その意味で限界はあるものの、国内で初めて臓器提供を行った 5 類型施設の臓器提供体制の具体的内容を明らかにした意義はある。今後は、質的にも調査し、臓器提供体制の望ましい在り方をさらに深める必要がある。

5．結論

ほぼ全ての脳死下臓器提供経験施設に院内 Co が設置されていた。臓器提供選択肢提示件数と臓器提供件数には有意な正の相関があった。院内 Co が 6 人以上又はポテンシャルドナーを把握する体制があると選択肢提示件数が多く、専従・専任院内 Co がいると脳死下及び心停止後臓器提供件数が有意に多かった。院内 Co は正確なポテンシャルドナーの把握と早期介入に寄与していると考えられた。院内 Co の適正な設置とポテンシャルドナー把握体制の構築が患者の臓器提供の意思を適切に汲み取り､臓器提供を完遂する体制整備に資すると考える。

利益相反

本論文に関して，開示すべき利益相反関連事項はない。

文献

1.公益社団法人日本臓器移植ネットワーク：脳死臓器移植の分析データ
https://www.jotnw.or.jp/data/brain-death-data.php
(2025 年 5 月 5 日閲覧)
2.公益社団法人日本臓器移植ネットワーク:日本で移植を受けられる割合.
https://www.jotnw.or.jp/explanation/07/03/.
(2024 年 12 月 25 日閲覧)
3.厚生労働省第 69 回厚生科学審議会疾病対策部会臓器移植委員会:臓器移植対策の現状について(2024 年 10 月 23 日)
https://www.mhlw.go.jp/content/10900000/001319467.pdf. (2024 年 12 月 25 日閲覧)
4. 厚生労働省第 70 回厚生科学審議会疾病対策部会臓器移植委員会: 今後の臓器移植医療のあり方について(2024 年 12 月 5 日)
https://www.mhlw.go.jp/content/10900000/001345699.pdf. (2025 年 5 月 5 日閲覧)
5.内閣府大臣官房政府広報室:移植医療に関する世論調査 世論調査報告書(令和 3 年 9 月調査)

https://survey.gov-online.go.jp/r03/r03-ishoku/. (2024 年 12 月 25 日閲覧)

6.久志本成樹:臓器提供病院への支援のあり方をどのようにするか.医学のあゆみ.2011; 237:466-470.

7.横田茉莉,安心院康彦,中原慎二,坂本哲也,横田裕行:脳死下臓器移植における医療者の負担感と支援ニーズに関する質問紙調査.日本救急医学会雑誌.2018;29:209-217.

8.中村俊介,有賀徹:脳死移植－提供側における諸問題－.日本消化器病学会雑誌.2011;108:729-734.

9. 佐々木聡,佐藤滋:献腎移植数増加のための院内体制整備のあり方の検討.日本臨床腎移植学会雑誌.2018;6:225-7.

10.厚生労働省.臓器提供施設連携体制構築事業について https://www.mhlw.go.jp/stf/seisakunitsuite/bunya/kenkou_iryou/kenkou/ishoku/renkeitaisei_00006.html. (2024 年 12 月 25 日閲覧)

11.厚生労働省第 65 回厚生科学審議会疾病対策部会臓器移植委員会:臓器移植対策の現状について(2023 年 11 月 15 日) https://www.mhlw.go.jp/content/10900000/001167551.pdf. (2025 年 5 月 5 日閲覧)

12.長谷川綾子,黒木雄一,小島加洋子,三浦清世美,佐野優太,小松智徳,絹川常郎,後藤百万.多職種連携型院内コーディネーター活動で得られた臓器提供数の増加.移植.2021;56:419-23.

13.中村晴美,縄田寛.家族の意思決定を支援する:院内ドナーコーディネーターの立場から.腎移植・血管外科.2023;35:71-6.

14.高橋絹代,小野元,古川博之,江川裕人:臓器提供可能医療機関の院内コーディネーター状況調査報告.移植.2020;55:119-24.

15.福嶌教偉:院内コーディネーターの体制整備と研修制度.Organ Biology.2015;22:31-8.

16.長谷川友紀,篠崎尚史,大島伸一:ドナーアクションプログラム—良質で確実な臓器提供をめざした院内体制の構築.医学のあゆみ. 2011;237:381-8.

17.Sadegh Beigee F, Mohsenzadeh M, Shahryari S, Mojtabaee M. Role of More Active Identification of Brain-Dead Cases in Increasing Organ Donation. *Exp Clin Transplant* 2017;15:60-2. DOI: 10.6002/ect.mesot2016.O42.

18.Hasanzade A, Nejatollahi SMR, Mokhber Dezfouli M, Hazrati M, Sheikholeslami S, Imani M, Mohseni B, Ghorbani F. The Impact of Early Brain-Dead Donor Detection in the Emergency Department on the Organ Donation Process in Iran. *Transpl Int* 2024;37:11903. DOI: 10.3389/ti.2024.11903.

19.Budoy D, Rodriguez-Villar C, DPeña D, Vizcaino F, Saavedra S, Bohils M, Quijada M, Cruz JM, Paredes-Zapata D, Ruiz A, Roque R, Garcia X, Bartolome RA. Effect of Active and Early Possible Organ and Tissue Donor Detection in the Emergency Room in a University Hospital. *Transplant Proc* 2019;51:3027-9. DOI: 10.1016/j.transproceed.2019.08.022.

20.平川達二,宮里均,大田守仁:沖縄県におけるドナーディテクションへの取り組み.腎移植・血管外科.2014;26:71-6.

21.小野元,田中雄一郎,加藤庸子:終末期における臓器提供意思を確認するための選択肢提示（オプション提示）方法の考察. 脳死・脳蘇生. 2020;32:46-51.

22.Elwyn G, Frosch D, Thomson R, et al: Shared decision making: a model for clinical practice. *J Gen Intern Med.* 2012;27:1361-7.

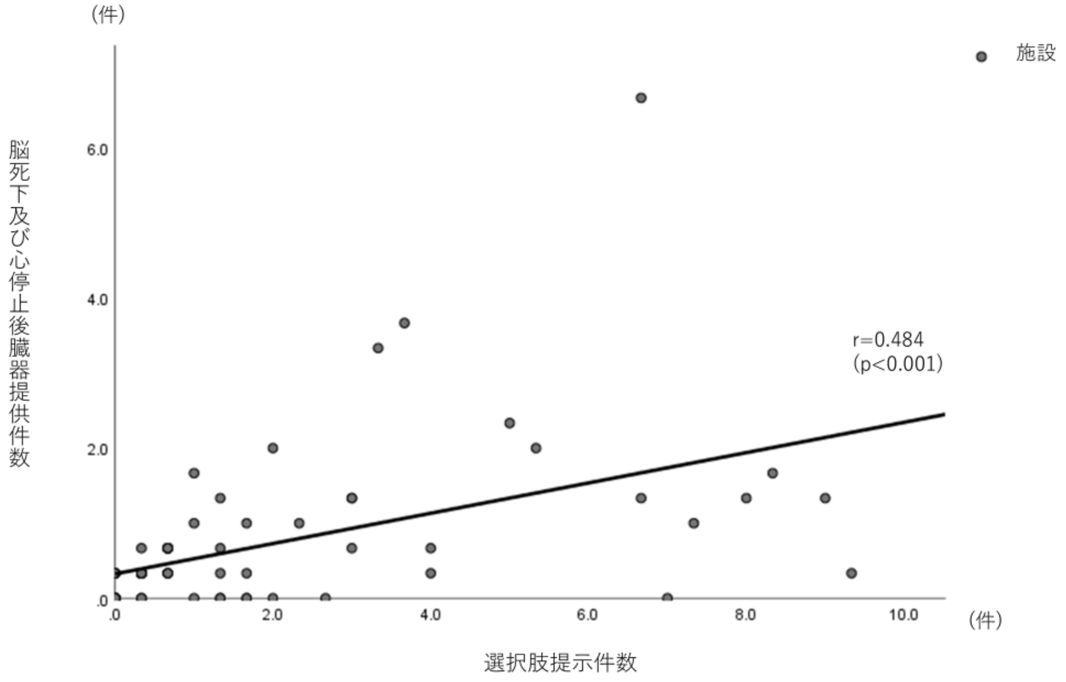

図1．選択肢提示件数と脳死下及び心停止後臓器提供件数（3年間の平均値）

表１．回答施設の属性(n=84) 平均±SD[range]

		n	%
地域	北海道	1	1.2%
	東北	2	2.4%
	関東甲信越	24	28.6%
	東海北陸	26	31.0%
	近畿	10	11.9%
	中国四国	8	9.5%
	九州沖縄	13	15.5%
病床数	500床未満	28	33.3%
	500床以上	56	66.7%
5類型施設の該当カテゴリー	救命救急センターとして認定された施設	58	69.0%
（複数回答）	日本脳神経外科学会の基幹施設又は連携施設	31	36.9%
	大学付属病院	25	29.8%
	日本救急医学会の指導医指定施設	24	28.6%
	日本小児総合医療施設協議会の会員施設	7	8.3%
臓器移植施設	臓器移植施設である	52	61.9%
	臓器移植施設でない	32	38.1%
年間救急搬送受入件数(n=80)		5,520.6±3,229.9[500-15,922]	
2023年度 脳死下臓器提供発生可能部署における死亡者数(n=60)		153.7±151.9[0-878]	
2023年度 脳死下臓器提供発生可能部署における脳死が見込まれた患者数(n=62)		8.5±9.1[0-50]	
2023年度 ポテンシャルドナー数(n=65)		6.0±7.8[0-37]	
2023年度 ポテンシャルドナーの関連部署への連絡件数(n=59)		3.4±4.6[0-19]	

※小数点第2位を四捨五入しているため、合計が100％にならないことがある。

表２．臓器提供体制整備状況(n=84)

		n	%
体制整備の内容	院内マニュアル整備	81	96.4%
（複数回答）	臓器提供関連委員会設置	72	85.7%
	脳死判定・臓器提供シミュレーション定期開催	57	67.9%
	移植医療研修会の定期開催	45	53.6%
	患者家族への普及啓発（ポスター掲示やパンフレット配布等）	60	71.4%
	患者意思確認システム（問診票等による意思確認システム等）	30	35.7%
	その他	1	1.2%
臓器提供施設連携体制構築事業への参加	参加している	45	53.6%
	参加していない	39	46.4%
「参加している」場合			
施設区分	拠点施設	14	31.1%
(n=45)	連携施設	31	68.9%
ポテンシャルドナーに選択肢提示を行う体制	ある	64	76.2%
	ない	20	23.8%
「ある」場合	担当医	62	96.9%
選択肢提示を行う職種	院内コーディネーター	20	31.3%
（複数回答）	担当看護師	3	4.7%
	その他	2	3.1%
選択肢提示の方法	口頭のみ	39	61.9%
	パンフレットのみ	9	14.3%
	口頭とパンフレットの併用	15	23.8%
ポテンシャルドナーを定期的に把握する活動	している	41	48.8%
	していない	43	51.2%

表3．院内コーディネーターの設置状況　(n=84)

		n	%	平均±SD[range
院内コーディネーターの設置	ある	81	96.4%	
	ない	3	3.6%	
1施設当たりの総数				7.0±6.0[1-36]
専従・専任院内Co	いる	16	19.0%	
	いない	65	77.4%	

表4．院内コーディネーターの職種別設置割合と１施設あたりの人数　(n=79　無回答を除く)

		%	平均±SD[range]
職種	看護師	97.5%	5.0±3.9[0-23]
	医師	49.4%	1.7±3.0[0-9]
	事務職員	26.6%	0.6±1.3[0-8]
	臨床検査技師	24.1%	0.4±0.9[0-4]
	その他	31.6%	0.4±0.8[0-4]

表5.　選択肢提示件数と脳死下及び心停止後臓器提供件数

		平均±SD	[range]	中央値
医療者から選択肢提示を行った件数	2021年度(n=68)	1.4±1.9	[0-7]	1.0
	2022年度(n=68)	1.8±2.7	[0-12]	1.0
	2023年度(n=71)	2.1±2.9	[0-12]	1.0
	3年間	1.9±2.5	[0-9.3]	0.7
脳死下及び心停止後臓器提供件数	2021年度(n=78)	0.6±0.8	[0-4]	0.0
	2022年度(n=81)	0.8±1.3	[0-6]	0.0
	2023年度(n=80)	0.8±1.4	[0-10]	0.0
	3年間	0.7±1.0	[0-6.7]	0.3

表6　臓器提供体制整備状況や院内Coの人数・種別による比較

		選択肢提示件数			脳死下及び心停止後臓器提供件数		
		n	中央値	p	n	中央値	p
連携事業参加	有	37	0.7	NS	43	0.3	NS
	無	34	0.7		38	0.3	
院内Co設置	有	68	0.7	NS	78	0.3	NS
	無	3	0.0		3	0.0	
院内Co人数	5人まで	39	0.3	0.003	45	0.3	NS
	6人以上	29	1.7		33	0.3	
専従・専任院内Co	有	13	0.7	NS	15	1.3	0.031
	無	55	0.7		63	0.3	
ﾎﾟﾃﾝｼｬﾙﾄﾞﾅｰ把握活動	有	34	1.3	0.022	39	0.3	NS
	無	37	0.3		42	0.3	
選択肢提示体制	有	55	0.7	NS	61	0.3	NS
	無	16	1.0		20	0.3	

Mann-Whitney’s U test
n：未回答施設を除く
